# Suggestions on the Contribution of Methyl Eugenol and Eugenol to Bay Laurel (*Laurus nobilis* L.) Essential Oil Preservative Activity through Radical Scavenging

**DOI:** 10.3390/molecules26082342

**Published:** 2021-04-17

**Authors:** Nikolaos Nenadis, Maria Papapostolou, Maria Z. Tsimidou

**Affiliations:** 1 Laboratory of Food Chemistry and Technology (LFCT), School of Chemistry, Aristotle University of Thessaloniki (AUTh), 57001 Thessaloniki, Greece; niknen@chem.auth.gr (N.N.); papaposm@chem.auth.gr (M.P.); 2 Natural Products Research Center of Excellence (NatPro-AUTH), Center for Interdisciplinary Research and Innovation (CIRI-AUTH), 57001 Thessaloniki, Greece

**Keywords:** essential oil, food preservatives, DFT, methyl eugenol, eugenol, bay laurel, radical scavenging, *Laurus nobilis*, structure-activity relationship

## Abstract

The present study examined the radical scavenging potential of the two benzene derivatives found in the bay laurel essential oil (EO), namely methyl eugenol (MEug) and eugenol (Eug), theoretically and experimentally to make suggestions on their contribution to the EO preservative activity through such a mechanism. Calculation of appropriate molecular indices widely used to characterize chain-breaking antioxidants was carried out in the gas and liquid phases (*n*-hexane, *n*-octanol, methanol, water). Experimental evidence was based on the DPPH^•^ scavenging assay applied to pure compounds and a set of bay laurel EOs chemically characterized with GC-MS/FID. Theoretical calculations suggested that the preservative properties of both compounds could be exerted through a radical scavenging mechanism via hydrogen atom donation. Eug was predicted to be of superior efficiency in line with experimental findings. Pearson correlation and partial least square regression analyses of the EO antioxidant activity values vs. % composition of individual volatiles indicated the positive contribution of both compounds to the radical scavenging activity of bay laurel EOs. Eug, despite its low content in bay laurel EOs, was found to influence the most the radical scavenging activity of the latter.

## 1. Introduction

Essential oils (EOs) from aromatic and medicinal plants display a wide range of biological properties, namely antibacterial, antifungal, and antioxidant [1,2,3,4], thus being good candidates as food preservatives [5]. The EOs’ antioxidant properties may be linked to the interaction with bacterial/fungal metabolism, which can cause oxidative spoilage (indirect antioxidant activity), and/or to the delay of lipid oxidation through the scavenging of formed free radicals (direct antioxidant activity) [6]. Their direct antioxidant activity has been mainly associated with the presence of chain-breaking antioxidants such as the phenols carvacrol, eugenol (Eug), or thymol [5,7]. Certain terpenoids, namely α-/γ- terpinene, α-phellandrene, etc., can also contribute by enhancing the free radical termination reactions (radical-radical coupling) [6].

The wide use of herbs and spices rich in EOs is an integral part of the Mediterranean diet [8]. Among them, there are some, either native Mediterranean (oregano, thyme) or non-native (clove) plants, the EOs of which are highly effective preservatives with significant radical scavenging potential owing to their abundance in phenolic compounds [7,9,10,11,12,13]. The EO obtained from bay laurel (*Laurus nobilis* L.), an evergreen tree or shrub native to southern Europe that is used not only as a flavoring agent in various dishes, food, and beverage preparations but also for its preservative properties [14,15,16], is characterized by a low content of phenolic type antioxidants. This leads to the hypothesis for a poor contribution to the EO preservative properties through a direct antioxidant activity mechanism. Specifically, this EO has been reported to contain only Eug in a concentration ranging from traces up to ~12.0% [17]. This phenol is much more active than other compounds reported to be present in abundance such as 1,8-cineole (typically, the major one), linalool, γ-terpinene, and ϐ-pinene [18]. Except for the aforementioned constituents, the EO contains also methyl eugenol (MEug), which is formed from Eug due to the activity of certain enzymes (e.g., *O*-methyltransferase) [19,20]. This compound, usually detected at a higher concentration than Eug [21,22], is a limiting factor for the use of bay laurel EO in food applications [23] due to its toxicity [24]. Although it lacks a free -OH moiety in the aromatic ring, some experimental data indicate that it presents radical scavenging activity [25,26,27]. Taking into consideration the above, in the present study, the radical scavenging potential of the two compounds was first examined theoretically via calculation of appropriate molecular indices widely used to characterize chain-breaking antioxidants [28]. Calculations were carried out in the gas and liquid phases aiming at approximating the activity in a solution (methanol and water) or bulk oils (*n*-hexane) and membrane lipids (*n*-octanol). For mechanistic purposes, other related compounds were also studied. Then, with the purpose to test experimentally the theoretical suggestions and hypothesis, the DPPH^•^ assay was applied to MEug, Eug, other main components (1,8-cineole, linalool, α-pinene), as well as to a set of chemically characterized with GC-MS/GC-FID bay laurel EOs. Our work aimed at contributing to the ongoing research on bay laurel EO as a source of multifunctional constituents for food and pharmaceutical applications.

## 2. Results and Discussion

### 2.1. Theoretical Evidence

**Figure 1:**
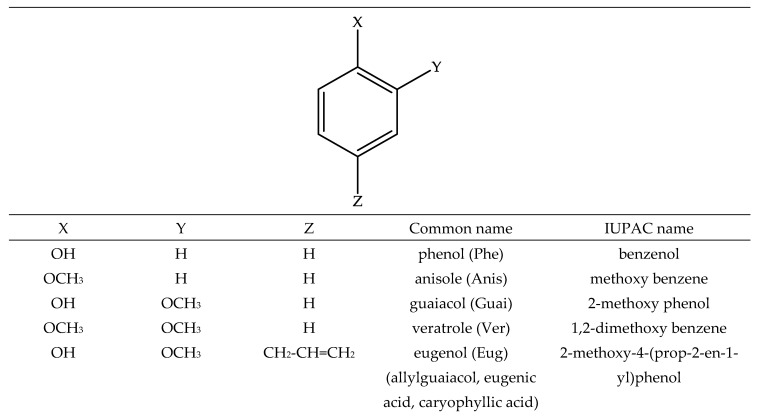

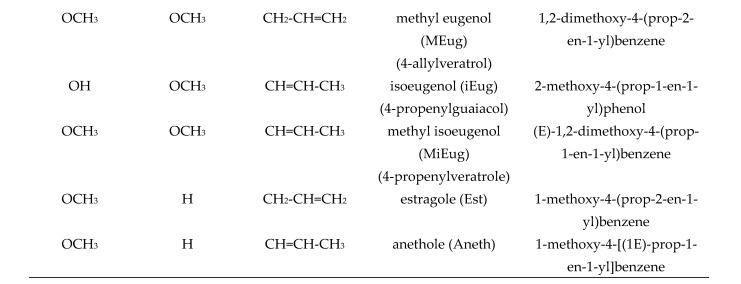
Structure of the test compounds.

To compute all the appropriate values of the corresponding molecular indices relevant to the radical scavenging activity, the most stable structure for MEug and the selected compounds (Figure 1) had to be located first. The optimized structures are provided as internal coordinates in the Supplementary Material (Table S1).

The selection of the 6-31G level was based on past studies for various phenolic compounds [29]. MEug was found non-planar. Although the methoxy group at C-4 in the input was set to be coplanar with the aromatic ring, after optimization, it was found to deviate from the plane by 126.217 Ǻ due to steric crowding. Such a conformation is different than that given by Chowdhry et al. [30] who, using spectroscopy (IR, Raman) and a different DFT approach, reported that the orientation of the two methoxy groups should be “*trans*” to minimize the steric hindrance. Examination of such a conformer with our approach showed that it can co-exist as its gas-phase enthalpy is only higher by 0.04 kcal/mol from the most stable structure we found. The side chain deviated from the plane by 50.16 Ǻ owing to the absence of extended conjugation when compared to iEug, MiEug, and Aneth. Similar observations were made for MEug in the liquid phase, where the corresponding angle was slightly higher (52.07 Ǻ in *n*-hexane; 55.01 Ǻ in *n*-octanol; 55.27 Ǻ in methanol; 55.49 Ǻ in water). The value of the dihedral angle formed by the carbon atoms of the side chain (122.29 Ǻ) was in close agreement with that reported in the literature (123.18 Ǻ) using a different computational approach for gas-phase calculations [30]. Non-planarity regarding the side chain (50.73 Ǻ) was also found for Eug and in agreement with our past study in the gas phase at 6-31 + G(d) [31] as well as for Est (51.09 Ǻ). As a consequence, MEug, Eug, and Est are expected to be located in the interphase of systems containing dispersed lipids such as liposomes, which is of high importance considering that it is the main site of oxidation [32]. MEug due to the presence of the second -OCH_3_ group becomes more lipophilic according to the Log *P* (logarithm of partition coefficient *P*) values (3.21 vs. 2.69) obtained theoretically (see Section 3.3). This was further supported by the dipole moment values obtained upon single-point energy calculations in the gas (2.1364 D vs. 2.5917 D) or the liquid phase (data not shown).

After locating the most stable conformation, appropriate molecular indices were calculated in the gas and liquid phases to characterize the scavenging of free radicals via the three prevailing mechanisms, namely hydrogen atom transfer (HAT), single-electron transfer followed by proton transfer (SET-PT), and sequential proton loss followed by electron transfer (SPLET). The examination was first carried out in the gas phase aiming at exploring the role of the structural characteristics without any external influence. Calculations in the liquid phase were carried out to simulate, as previously mentioned, real systems. To get insight into the role of structural features in the predicted activity, except for Phe, which was selected as a reference compound with no or negligible activity, other structurally related compounds, most of which are constituents of natural EOs, were included (Figure 1). The computed indices in the gas phase are given in Table 1. For SET-PT and SPLET mechanisms, the values of ionization potential (IP) and proton affinity (PA) are only provided considering that these values are critical to highlight whether test compounds can follow any of these two mechanisms. In the same table, the relative difference (Δ) of each value to that computed for Phe (reference) is also provided for better evaluation of the activity.

#### 2.1.1. Hydrogen Atom Donation

**Figure 2:**
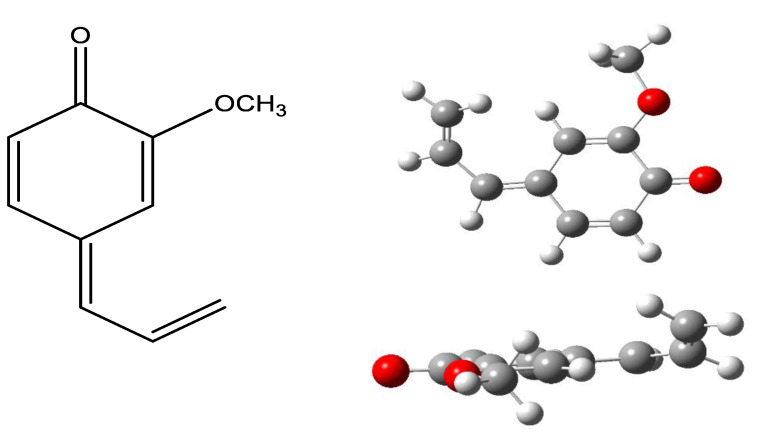
Derived quinone from Eug after the donation of two hydrogen atoms following a step-wise hydrogen atom transfer (gas phase at B3LYP/6-31G).

As shown, based on the gas-phase BDE values presented in Table 1, it was evident that MEug, though lacking an -O-H group in the aromatic ring, could act as a hydrogen atom donor through the abstraction of an allyl hydrogen atom located in the side chain. The computed BDE value for the corresponding C-H bond (72.7 kcal/mol) was low, as a consequence of the influence of the aromatic ring and the double bond at the end of the side chain. The value was almost the same as that for the equivalent bond in Eug (72.3 kcal/mol) showing that it was not influenced by the substitution pattern in the aromatic ring. This was further verified by the -C-H BDE value calculated for Est (72.5 kcal/mol) lacking an -O-H (Eug) at C-4 or an -OCH_3_ (MEug) substituent at *o*-position and the published BDE values for Est and Eug in the liquid phase (72.7 and 72.8 kcal/mol, respectively) [18]. Lower BDE value by 10.1 kcal/mol of the C-H bond to that of the O-H one in Eug suggests that the compound should preferentially donate first a hydrogen atom from the side chain. A similar observation has been already made theoretically in the past by other authors who reported a lower difference in the corresponding BDE values (6–8.6 kcal/mol) in the gas or liquid phase [18,33]. The donation of the hydrogen atom from the -O-H group can follow during sequential HAT. Such a donation should be much easier as the BDE value becomes significantly lower (66.6 kcal/mol) due to the formation of a fully conjugated compound that is very stable (Figure 2).

The latter surprisingly was not completely planar as the end of the side chain deviated by 32.5 Ǻ. Nevertheless, this seems to be the most stable structure as the repeating of the optimization process using an input with a complete planar structure resulted in the same structure depicted in Figure 2. Such a finding was different from that reported for a catechol derivative, namely dihydro-caffeic acid, for which it was shown computationally in the gas phase that the allyl hydrogen atom could only be abstracted after the formation of an *o*-quinone [28]. In terms of the presented evidence, it could be hypothesized that MEug is an efficient hydrogen atom donor, though less potent than Εug as the latter could donate two hydrogen atoms.

#### 2.1.2. Electron Donation

Electron donation is favored in polar media as the process, regardless of SET-PT or SPLET, leads to charge separation. In the first mechanistic route, IP is the rate-determining parameter. In terms of the corresponding computed values, as well as that of the compounds examined for comparison, it was also evident that all the introduced structural features to MEug contributed to the lowering of the IP value by 25.0 kcal/mol compared to Phe (Table 1). More specifically, the introduction of a -OCH_3_ in the aromatic ring (Guai) or replacement of a phenolic -O-H group by an -OCH_3_ (Anis) favored the electron donation more. Both these features should account for the significant lowering of IP value in MEug considering the finding for Ver. Less was expected to be the contribution of the side chain as the further reduction by comparing the IP values of MEug and Ver was calculated to be only 3.8 kcal/mol. Similar were the findings for Eug (reduction by 6.4 kcal/mol) comparing the IP value to that of Guai. As also observed for HAT, the influence of the prop-2-en-1-yl chain was lower than that observed for those compounds with extended conjugation (prop-1-en-1-yl) in the side chain of the same length (the IP values of iEug, MiEug, and Aneth were 9, 8, and 11.4 kcal/mol lower than that of Eug, MEug, and Est, respectively). This is further supported by the difference (14.5 kcal/mol) reported in the literature for Aneth and Est according to the vertical ionization potential values in the aqueous phase [18]. This highlights the importance of this feature for efficient electron donation. Based on the obtained values, MEug was predicted to be a better electron donor than Eug. It should be mentioned, though, that taking into account the observations made by Wright et al. [34], none of these compounds should act via SET as the computed gas-phase ΔIP values, even the larger ones for iEug and MEug, were lower than those reported for tocopherols (30.5–36.1 kcal/mol) for which mechanistic studies indicate that HAT is the preferable route of action. Therefore, there was no need to calculate proton-dissociation enthalpy values characterizing the second step of SET-PT. The second mechanistic route, SPLET, was not feasible because the formation of anions was not expected according to the calculated proton affinity values (PA) in the gas phase, which were found to be higher than that of Phe (348.0 kcal/mol) or negligibly lower (−0.5 kcal/mol for iEug). The case of the compounds lacking an -O-H group formation of anions through ionization of a carbon atom was expected to be even less possible in terms of gas-phase PA values (360.9–364.6 kcal/mol). This is in accordance with the PA values computed in the gas and aqueous phase by Boulebd [33] for the C-H bond of the allyl hydrogens and the O-H one in Eug. Consequently, electron transfer enthalpy (ETE) values were not computed.

#### 2.1.3. Solvent Effect

Calculations were then carried out taking into account explicit solvent effect aiming at approximating real systems. Only in the case of *n*-hexane, simulating bulk oils, the computation was limited to BDE values, as the formation of ionic species is not facilitated in such an environment.

##### Hydrogen Atom Transfer

The BDE values in the four selected solvents are given in Table 2. Based on the BDE values for the test compounds bearing labile -O-H and/or -C-H, the same trend in activity was observed in all solvents. The C-H BDE values remained lower than those of the -O-H and, in agreement with gas-phase findings, slight was the influence of aromatic ring substitution. MEug was found to be a potent hydrogen atom donor with the activity estimated to be higher according to the calculated ΔBDE values in comparison to that obtained in the gas phase. The contribution of an -OCH_3_ at *o*-position was more pronounced, especially in more polar environments as Guai, Eug, and iEug were also more potent than in the gas phase. The extension of conjugation in the side chain (iEug) reduced the BDE value by 2.7–3.0 kcal/mol.

##### Electron Donation

The IP values for all the test compounds are presented in Table 3. As evident in comparison to the gas-phase ones, the introduction of the explicit solvent effect caused a reduction in the values, with a larger effect in more polar media, in accord with the findings of Boulebd [33] for various EO components, including Eug and iEug.

In terms of the corresponding computed values, it was evident that MEug was a less potent electron donor in comparison to the gas-phase study as the ΔIP values were in the range −9.5 to −11.9 kcal/mol, significantly lower than in the former (−25.0 kcal/mol). Contrary to gas-phase calculations, the replacement of an -O-H group by an -OCH_3_ at C-4 reduced the electron donation activity, as the IP value was higher in all solvents than that of Phe. Introduction of a second -OCH_3_ in *o*-position did not have a clear positive effect. Thus, in all solvents, MEug was found slightly less active than Eug as the corresponding IP values were higher by 1.9 to 3.1 kcal/mol. The same reverse trend was observed for the pair iEug-MiEug. The finding for Aneth-Est indicating the superiority of the extended conjugation was in line with gas-phase data. Taking into account that the ΔΙP values of the various compounds were smaller than those calculated in the gas phase indicates that lower is the possibility of an electron-donating mechanism even in the polar media. The second mechanistic route, SPLET, was rather not probable granted that the calculated PA values were found to be higher than that of phenol for most of the compounds (Table 4). In those cases that the PA values were lower, the difference was negligible (≤1.0 kcal/mol). The ionization of -O-H was much easier than that of the C-H.

### 2.2. Experimental Evidence

The scavenging activity of the DPPH radical (DPPH^•^) by pure MEug was examined comparatively to that of pure Eug in methanol to test experimentally the theoretical findings. The determination of IC_50_ values showed that MEug had some activity but low (IC_50_ = 80 [MEug]/[DPPH^•^], mol/mol) in comparison to that of Eug (IC_50_ = 0.3 [Eug]/[DPPH^•^], mol/mol). This result is in general agreement with that of other publications employing the DPPH^•^ assay [25,35,36]. The magnitude of the reported difference varies (Eug vs. MEug: 1.23-fold up to >21-fold more efficient). Joshi [37] reported MEug as inactive toward DPPH^•^; however, this could be related to both the short monitoring period (20 min) of the reaction and the low [AH]/[DPPH^•^] ratio selected upon testing (0.004–0.032). As the C-H BDE values for both MEug and Eug were found to be comparable, and electron donation is expected to have little influence on their activity, the experimental findings for MEug (higher RSA% values when prolonging reaction monitoring to 20 h) indicate that if an allyl hydrogen atom donation takes place, this occurs at a rather slow pace. The observation is further supported by Amorati et al. [6] who, working on literature data, found that α-tocopherol (O−H BDE value 77.2 kcal/mol) reacted by 10^6^-fold faster with DPPH^•^ than 1,4-cyclohexadiene (C-H BDE value 77.9 kcal/mol). The explanation given by Denison and Denisova [38] about the reaction of aminyl radicals with the C-H bonds of alkylperoxyl radicals or the O-H bonds of hydroxyperoxyl radicals could account for the aforementioned observations. Specifically, the argument that the reaction with O-H bonds is fast due to “virtually no activation energy”, which is associated with the triplet repulsion in the two reaction centers (C…H…N vs. O…H…N) and the difference in the electronegativities of the interacting atoms. Therefore, despite the lower C-H BDE value in Eug the hydrogen atom donation should be favored due to the presence of the O-H group. The much higher activity should eventually be related to the donation of its allyl hydrogen as well, through a sequential HAT, leading to the formation of the compound depicted in Figure 2. The latter suggestion could be supported by the higher RSA% values observed for Eug when prolonging the reaction monitoring from 1 to 20 h.

Next, experimental evidence was sought for bay laurel EOs. The DPPH^•^ scavenging activity expressed as Trolox or α-tocopherol equivalents, and the % concentration of the most abundant components identified are given in Table 5. The EOs differed in the concentration of 1,8-cineole widely (34.1–63.0%), which is in line with what has been previously reported for this constituent [15,22]. Similarly, the EOs differed in their percent composition in MEug, Eug, and their ratio, which is also backed by the literature [39]. Considering that traceability is an issue in commercial EO samples, such composition variations are expected and can be attributed to differences in, among others, the origin of plant material [22,40], the plant organs used [39,41,42], and also to the extraction methods applied [43]. The presence of Eug was found to be affirmative for assigning radical scavenging activity to bay laurel EO as evidenced via Pearson correlation analysis between antioxidant activity values and the % concentration values of the EOs’ major constituents. Results show the highest correlation for Eug (*r* = 0.916, *p* = 0.000). MEug followed (*r* = 0.785, *p* = 0.000), despite the ~270-fold lower activity than that of pure Eug. Other compounds indicated to contribute less were linalool (*r* = 0.587, *p* = 0.007) and α-terpinyl acetate (*r* = 0.484, *p* = 0.031). Pure linalool, however, was not found active even after 20 h of reaction with the radical upon testing at a high concentration ([linalool]/[DPPH^•^], mol/mol = 90). Such an observation is corroborated by the high IC_50_ value of the compound using the DPPH^•^ assay and the higher BDE value by ~6.5 kcal/mol compared to that of Eug according to literature data [18]. α-Pinene, not correlating with the antioxidant activity values, was also found inactive. The same was evidenced for 1,8-cineole, the most abundant EO compound when examined at a high concentration ([1,8-cineole]/[DPPH^•^], mol/mol = 90), even after 20 h of reaction monitoring. The lower activity of 1,8-cineole even than that of MEug is in agreement with the work of Ballesteros et al. [44], who reported a 2-fold lower potency toward the DPPH^•^ scavenging for the former. The high BDE values computed in the present study in methanol for different C-H bonds (88.0–93.0 kcal/mol) of the compound and, as verified by published data in the aqueous phase [18], may justify this finding. The corresponding monoterpenoid, except for being rather inactive, had a negative influence in DPPH^•^ scavenging activity of the EOs as its levels were negatively correlated (*r* = −0.642, *p* = 0.002) with the Trolox equivalent values. A similar trend to that has been reported by Chrysargyris et al. [45] upon studying various aromatic and medicinal plants with DPPH^•^ including a set of laurel EOs. The authors attributed that negative correlation to its low activity. However, such a trend could also be linked to a molecular crowding effect due to the high concentration of 1,8-cineole. As a consequence, the access to the free radical by other active compounds present at lower concentration is hindered [46], with the effect expected to be more pronounced at higher 1,8-cineole levels of concentration. A multivariate approach (partial least square regression, PLS-R) that takes into account the concomitant contribution of the concentration of EOs’ components to the antioxidant activity values verified the observations made by Pearson correlation for MEug, Eug, and 1,8-cineole via a statistically significant model (*F* = 81.71, *p* = 0.000, *r*^2^ = 0.956, *r*^2^_(prediction)_ = 0.820). Thus, the standardized coefficient obtained for Eug was the highest (2.28903), ~8.3-fold higher than that of MEug (0.27670), whereas that of 1,8-cineole was negligible (0.00227).

Conclusively, theoretical suggestions indicated that MEug may contribute to the preservative activity of bay laurel EO through radical scavenging via allyl hydrogen atom donation. Such a contribution should be lower than that of Eug which may donate two hydrogen atoms following a sequential HAT mechanism. Experimental evidence supported theoretical suggestions and additionally indicated that Eug despite being found at low concentration in bay laurel EO influences the most its radical scavenging efficiency. Our findings contribute to the knowledge for MEug and Eug radical scavenging activity per se or as constituents of bay laurel EO.

## 3. Materials and Methods

### 3.1. EO Samples, Standards, and Chemicals

Commercial bay laurel EO samples (*n* = 20), namely OL1-3, AL1-3, SL1-3, FL1-3, EL1-2, VL2, HL, DL, AmL, ML, and TL, were purchased from producers, herbal shops, and pharmacies (Thessaloniki, Greece). All products already packed in amber glass vials were stored at 4 °C until analysis. 2,2′-diphenyl-1-picrylhydrazyl (DPPH), 6-hydroxy-2,5,7,8-tetramethylchroman-2-carboxylic acid (Trolox), α-tocopherol (≥96%), and the standards α-pinene (98%), 1,8-cineole (food-grade, ≥99%), MEug (food-grade, ≥98%), and Eug (99%) were from Sigma-Aldrich (Sigma-Aldrich Chemie GmbH, Steinheim, Germany); (±)-linalool (GC, ≥95%) was obtained from Fluka (Fluka Chemie GmbH, Buchs, Switzerland); HPLC-grade methanol was purchased from VWR (VWR chemicals, Fontenay-sous-Bois, France) and GC-grade dichloromethane (≥99.9%) from Honeywell (Charlotte, NC, USA).

### 3.2. Theoretical Calculations

All calculations for tested compounds were performed by the Gaussian 09W rev. A.02- SMP program [47]. The B3LYP functional was used for geometry optimization and computation of harmonic vibrational frequencies using the 6-31G basis set (unrestricted B3LYP for the resulting phenoxy and cation radicals). Single-point electronic energies were then obtained using the 6-311++G(2d,2p) basis set, including dipole moment values (Debye, D). Employing the electronic energies (298 K) at 6-311++G(2d,2p) and thermal contributions to enthalpy obtained at 6-31G, the bond dissociation enthalpy (BDE) values that characterize hydrogen atom transfer (HAT) activity were determined according to the equation:

(1) BDE= Hr + Hh − Hp

where Hr is the enthalpy of the radical generated by H atom abstraction, Hh is the enthalpy of the H atom, and Hp is the enthalpy of the parent molecule.

The adiabatic ionization potential (IP) values that characterize the first step of the single electron−proton transfer (SET-PT) mechanism were determined according to the equation:

(2) IP = Hcr − Hp

where Hp and Hcr stand for the enthalpy of the parent molecule and the corresponding cation radical generated after electron transfer.

Proton affinity (PA) values that define the first step of the SPLET (sequential proton loss electron transfer) mechanism were calculated according to the formula:

(3) PA = Ha + Hpr − Hp

where Ha is the enthalpy of the anion generated after deprotonation, Hpr is the enthalpy of the proton, and Hp is the enthalpy of the parent molecule.

No spin contamination was found for radicals, the total spin operator ⟨S2⟩ values being about 0.750 in all cases, suggesting that the used wavefunctions were reliable regarding geometries, energies, and population analyses. Implicit solvent effects (water) were taken into account with the aid of integral equation formalism of the polarized continuum model (IEF-PCM) and the united atom for Hartree−Fock (UAHF) solvation radii [48,49]. Structure optimization of the parent molecules and derived species was carried out in the liquid phase using the above model at the 6-31G level of theory. Then single-point electronic energies were obtained using the 6-311++G(2d,2p) basis set. All the computed values of thermodynamic molecular descriptors were expressed in kcal/mol (1 Hartree = 627.509 kcal/mol).

### 3.3. Estimation of Partition Coefficient (Log P)

Calculation of the Log *P* values, which express the partitioning of the phenols in an *n*-octanol/water system, was based on the CS ChemDraw Ultra and chemical structure drawing standard 1985–2003 program (CambridgeSoft Corporation, Cambridge, MA, USA). The values used relied on Broto’s fragmentation method [50].

### 3.4. DPPH^•^ Assay

The antioxidant activity of bay laurel EOs, as well as that of standards, was determined using the DPPH^•^ assay according to Sellami et al. [51] with some modifications. A radical solution was prepared in methanol at a concentration of 0.06 mM. Methanolic solutions of standard compounds were prepared in a range of concentrations namely 0.5–6 mM for Eug, 4–400 mM for MEug, α-pinene, 1,8-cineole, and linalool, whereas those of the EOs were at 5 mg/mL. An aliquot of 40 and 100 μL of standards and EOs solutions, was mixed with 2960 and 2900 μL of DPPH^•^ solution, respectively. After mixing for 20 s using a vortex, the absorbance of the test solutions was recorded at 515 nm with a spectrophotometer (Shimadzu UV 1601, Kyoto, Japan) after storage in the dark at room temperature for 1 h. The absorbance of standards solutions was also recorded after 20 h. Each measurement was carried out in triplicate and appropriate blanks were used for correction. The repeatability of the procedure was checked (CV% = 5, *n* = 5). Radical scavenging activity % (RSA%) was calculated using the following equation:

(4) RSA% =100×[(Abs blank (t=30))−(Abs sample)]/[(Abs blank (t=30))]

The concentration of MEug and Eug that caused 50% radical scavenging (IC_50_) was calculated by plotting the RSA% values vs. the corresponding [AH]/[DPPH^•^], mol/mol, ratio. RSA% values obtained for the EOs were converted to µmol Trolox or α-tocopherol per mg EO via the construction of 5-point standard curves using solutions of 0.1–0.55 mM (0.06–0.32 [AH]/[DPPH^•^], mol/mol).

### 3.5. GC-FID and GC-MS Analyses of EOs

The GC-FID analyses were accomplished with an Agilent 6890A gas chromatograph equipped with a split-splitless injector. Samples were analyzed on a TR-FAME capillary column (60 m × 0.25 mm i.d., film thickness 0.25 μm) (Thermo Scientific, Bellefonte, PA, USA). Helium was used as carrier gas at a constant flow rate (2 mL/min). Samples were diluted in dichloromethane 2% (*v/v*) and then injected (2 μL) manually onto the GC in split mode with a 25:1 ratio. The injector and detector were both kept at 240 °C. Separation conditions used were as follows: oven temperature was initially set at 40 °C for 5 min, then raised to 100 °C at 15 °C/min, then to 140 °C at 5 °C/min, held for 1 min, and finally raised at 240 °C at 15 °C/min and kept for 5 min. All analyses were performed in duplicate. The repeatability was checked (CV% = 2.9, *n* = 5). For the GC-MS analyses, an Agilent 6890A gas chromatograph (GC) was employed equipped with a Mass Selective Detector MSD 5973 mass spectrometer (Agilent Technologies, Palo Alto, CA, USA) and a DB-WAX capillary column (polyethylene glycol: 30 m × 0.25 mm i.d., 0.33 µm film thickness) (Agilent Technologies, Palo Alto, CA, USA). The carrier gas, sample preparation, injection, and separation conditions were as previously described. The transfer line temperature was set at 240 °C. MS conditions were as follows: ionization energy, 70 eV; electronic impact ion source temperature, 230 °C; scan rate, 2.36 scan/s; mass range, 35–350 amu. The solvent delay time was set at 7 min. Selected volatile constituents were identified by comparing their retention time (t_R_) with that of standard compounds and their mass spectra with data from the NIST library (Version 2.0f, National Institute of Standards and Technology, Gaithersburg, MD, USA, 2008) and published literature. In addition, retention indices (RIs) were determined relative to the t_R_ of series of *n*-alkanes (C_8_–C_30_).

### 3.6. Statistical Analysis

Statistical comparisons of the mean antioxidant activity values of EOs were performed by one-way ANOVA, followed by the multiple Duncan test (*p* ≤ 0.05 confidence level) using SPSS 20.0 (SPSS Inc., Chicago, USA). Pearson correlation and PLS-R (employing leave-one-out cross-validation) were carried out with the aid of Minitab 17 (Minitab Inc., State College, PA, USA).

## Figures and Tables

**Table 1:** Absolute and relative (Δ) BDE, (Δ) ionization potential (IP), and (Δ) proton affinity (PA) values (kcal/mol) of the test compounds in the gas phase at B3LYP/6-31G//B3LYP/6-311++(2d,2p) level.

	BDE	ΔΒDE ^1^	IP	ΔIP ^1^	PA	ΔPA ^1^
AH	kcal/mol					
Phe	84.3	0.0	191.8	0.0	348.0	0.0
Anis	-	-	185.1	−6.7	-	-
Guai	83.4	−0.9	177.8	−14.0	352.7	4.8
Ver	-	-	170.6	−21.2	-	-
MEug	72.7 ^2^	−11.6	166.8	−25.0	360.9 ^2^	12.9
Eug	82.4/72.3 ^2^	−1.9/−12.0	171.4	−20.4	351.4/364.1 ^2^	3.4/16.1
MiEug	-	-	158.8	−33.0	-	-
iEug	79.2	−5.1	162.4	−29.4	347.5	−0.5
Est	72.5 ^2^	−11.8	176.8	−15.0	364.6 ^2^	16.6
Aneth	-	-	165.4	−26.4	-	-

^1^ value of the test compound − value of Phe; ^2^ refer to allyl -C-H bond.

**Table 2:** Absolute and relative (Δ) BDE values (kcal/mol) of the test compounds with labile -O-H and/or C-H bonds computed in *n*-hexane, *n*-octanol, methanol, and water at B3LYP/6-31G//B3LYP/6-311++(2d,2p) level.

Solvent	*n*-Hexane		*n*-Octanol		Methanol		Water	
	BDE	ΔBDE ^1^	BDE	ΔBDE ^1^	BDE	ΔBDE ^1^	BDE	ΔBDE ^1^
AH	kcal/mol							
Phe	86.3	0	88.5	0	89.2	0	89.1	0
Guai	83.8	−2.5	83.7	−4.8	83.7	−5.5	83.6	−5.5
MEug	74.1 ^2^	−12.2	75.7 ^2^	−12.8	76.0 ^2^	−13.3	76.1 ^2^	−13.0
Eug	82.3/73.5 ^2^	−4.0/−12.8	81.6/74.9 ^2^	−6.9/−13.6	80.9/75.4 ^2^	−8.3/−13.9	81.3/75.2 ^2^	−7.8/−13.9
iEug	79.3	−7.0	78.6	−9.9	78.4	−10.8	78.3	−10.8
Est	73.7 ^2^	−12.6	75.2 ^2^	−13.3	75.5 ^2^	−13.8	75.6 ^2^	−13.5

^1^ value of the test compound − value of Phe; ^2^ refer to allyl -C-H bond.

**Table 3:** Absolute and relative (Δ) IP values (kcal/mol) of the test compounds in *n*-octanol, methanol, and water at B3LYP/6-31G//B3LYP/6-311++(2d,2p) level.

Solvent	*n*-Octanol		Methanol		Water	
	IP	ΔIP ^1^	IP	ΔIP ^1^	IP	ΔIP ^1^
AH	kcal/mol					
Phe	139.4	0.0	134.9	0.0	133.2	0.0
Anis	140.1	0.7	136.6	1.7	135.7	2.5
Guai	130.4	−9.0	126.4	−8.5	125.4	−7.8
Ver	130.2	−9.2	127.1	−7.8	126.3	−6.9
MEug	127.5	−11.9	124.4	−10.5	123.7	−9.5
Eug	125.6	−13.8	121.6	−13.3	120.6	−12.6
MiEug	123.1	−16.3	120.3	−14.6	119.6	−13.6
iEug	121.0	−18.4	117.5	−17.4	116.6	−16.6
Est	133.2	−6.2	129.6	−5.3	128.7	−4.5
Aneth	125.6	−13.8	122.5	−12.4	121.7	−11.5

^1^ value of the test compound-value of Phe.

**Table 4:** Absolute and relative (Δ) PA values (kcal/mol) of the test compounds in *n*-octanol, methanol, and water at B3LYP/6-31G//B3LYP/6-311++(2d,2p) level.

Solvent	*n*-Octanol		Methanol		Water	
	PA	ΔPA ^1^	PA	ΔPA ^1^	PA	ΔPA ^1^
AH	kcal/mol					
Phe	301.6	0.0	297.8	0.0	296.5	0.0
Guai	302.0	0.4	297.2	−0.6	295.9	−0.6
Eug	302.6/326.8 ^2^	1.0/25.2	297.7/323.7 ^2^	−0.1/26.7	296.5/322.9 ^2^	0/26.5
MEug	324.7 ^2^	23.1	321.8 ^2^	24.0	321.0 ^2^	24.5
iEug	301.4	−0.2	296.8	−1.0	295.6	−0.9
Est	327.4 ^2^	25.8	324.4 ^2^	26.6	323.5 ^2^	27.0

^1^ value of the test compound − value of Phe; ^2^ refer to allyl -C-H bond.

**Table 5:** Antioxidant activity of bay laurel essential oils (EOs) expressed as Trolox (or α-tocopherol) equivalents, GC-FID percent composition (%) of their most abundant components, and MEug/Eug ratio.

EO	Antioxidant Activity (*n* = 3)	Most Abundant Volatiles (GC-MS Analyses ^1^)								Ratio
	μmol Trolox/mg EO (μmol α-Tocopherol/mg EO)	α-Pinene	Limonene	1,8-Cineole	Linalool	Terpinen-4-ol	α-Terpinyl Acetate	MEug	Eug	MEug/Eug
		RI ^2^								
		1043	1120	1159	1456	1584	1683	2082	2338	
		*m/z* ^3^								
		*n*.a. ^4^	68, 93, 136	43, 81, 154	41, 71, 153	71, 111, 154	43, 121, 181	147, 163, 178	103, 149, 164	
		Percent Composition (%)								
OL1	4.4 ± 0.0 ^g^ (4.8 ± 0.0 ^g^)	6.0	0.9	61.9	2.8	3.4	9.1	1.1	0.8	1.38
OL2	5.6 ± 0.0 ^h^ (6.0 ± 0.0 ^h^)	7.1	1.6	51.0	3.0	2.8	14.0	1.7	1.2	1.42
OL3	4.7 ± 0.1 ^g^ (5.0 ± 0.1 ^g^)	5.6	2.1	58.4	0.9	2.5	14.2	1.1	0.8	1.38
AL1	8.1 ± 0.1 ^i^ (8.4 ± 0.1 ^i^)	-	-	34.1	10.6	6.1	28.0	7.6	2.1	3.62
AL2	3.6 ± 0.0 ^c^ (4.0 ± 0.0 ^c^)	2.5	3.5	48.6	1.8	4.3	16.1	1.3	1.5	0.87
AL3	3.9 ± 0.2 ^abd^ (4.3 ± 0.2 ^abd^)	7.1	2.9	59.5	3.3	1.6	8.9	2.5	0.5	5.00
SL1	7.7 ± 0.2 ^k^ (8.0 ± 0.2 ^k^)	6.4	2.2	58.2	6.1	1.6	8.8	2.2	2.1	1.05
SL2	3.6 ± 0.1 ^ac^ (4.0 ± 0.1 ^ac^)	7.5	2.6	58.0	3.5	2.2	8.9	2.2	0.5	4.40
SL3	4.0 ± 0.1 ^bd^ (4.4 ± 0.1^bd^)	7.5	2.7	59.1	3.5	1.9	8.2	2.2	0.5	4.40
FL1	3.8 ± 0.3 ^abc^ (4.2 ± 0.3 ^abc^)	6.4	2.3	59.6	3.5	2.3	10.2	2.2	0.5	4.40
FL2	4.2 ± 0.2 ^d^ (4.5 ± 0.2 ^d^)	8.2	2.7	54.7	3.6	1.7	10.4	2.6	0.6	4.33
FL3	3.9 ± 0.1 ^abd^ (4.3 ± 0.1 ^abd^)	7.0	2.5	59.1	3.5	1.5	9.7	2.8	0.5	5.60
EL1	3.0 ± 0.1 ^f^ (3.4 ± 0.1 ^f^)	6.7	2.5	63.0	3.8	1.9	9.3	1.0	0.4	2.50
EL2	5.8 ± 0.0 ^h^ (6.1 ± 0.0 ^h^)	2.9	1.3	53.6	6.7	2.8	13.2	5.2	1.2	4.33
VL2	8.1 ± 0.3 ^i^ (8.4 ± 0.3 ^i^)	3.6	2.3	47.7	12.9	1.3	14.0	8.0	2.1	3.81
HL	2.4 ± 0.0 ^e^ (2.8 ± 0.0 ^e^)	7.4	2.2	61.4	3.6	1.3	10.7	1.1	0.4	2.75
DL	3.2 ± 0.2 ^f^ (3.7 ± 0.2 ^f^)	8.6	3.1	57.7	3.5	1.6	8.7	2.1	0.4	5.25
AmL	2.5 ± 0.1 ^e^ (2.9 ± 0.1 ^e^)	3.2	1.4	53.2	10.7	-	13.2	-	0.3	0.00
ML	2.1 ± 0.3 ^j^ (2.6 ± 0.3 ^j^)	3.2	1.6	61.2	1.4	2.4	12.9	0.8	0.3	2.67
TL	3.7 ± 0.0 ^abc^ (4.1 ± 0.0 ^abc^)	7.1	3.0	58.4	3.3	1.8	8.6	2.5	0.5	5.00

^1^ Chromatograms are provided in Figure S1; ^2^ RI—experimental retention index on a polar TR-FAME column; ^3^ two main peaks and the molecular one; ^4^ not applicable, compounds eluting before 7 min were not detected due to solvent delay; values with different lower-case letters are statistically different at *p* < 0.05.

## Data Availability

The data presented in this study are available in supplementary material.

## Supplementary Materials

The following are available online. Table S1: Internal coordinates of the optimized structures of the test compounds in the gas-phase at B3LYP/6-31G, Figure S1: GC-MS chromatograms of bay laurel EO samples. Peaks 1–7 were cross-referenced against the NIST mass spectral library (version 2.0f, 2008) and assigned to (1) limonene, (2) 1,8-cineole, (3) linalool, (4) terpinen-4-ol, (5) α-terpinyl acetate, (6) MEug, and (7) Eug.

## Sample Availability

Samples are not available from the authors.
