# Supplementary material for: Suggestions on the Contribution of Methyl Eugenol and Eugenol to Bay Laurel (Laurus nobilis L.) Essential Oil Preservative Activity through Radical Scavenging
Source: Molecules. 2021 Apr 17;26(8):2342. doi: 10.3390/molecules26082342 (PMC8073261; doi:10.3390/molecules26082342)
Supplement: Supplementary file 1 [file molecules-26-02342-s001.zip › molecules-1166306-supplementary.pdf]

Table S1. Internal coordinates of the optimized structures of the test compounds at B3LYP/6-31G in the gas-phase

**Phe**
C,0,-2.0146131067,0.755600187,-0.2153405328<br>C,0,-1.5232934102,-0.5105125795,0.1234803932<br>C,0,-2.4033679746,-1.5539294409,0.4302255526<br>C,0,-3.7857574695,-1.3226171178,0.3960224475<br>C,0,-4.2877464583,-0.063749848,0.0594563599<br>C,0,-3.3976010516,0.9724775415,-0.2455290193<br>O,0,-3.9592846739,2.20514627,-0.5736790719<br>H,0,-1.3280124828,1.5642558944,-0.4532140233<br>H,0,-0.4508924174,-0.6773814215,0.1465438618<br>H,0,-2.0187350017,-2.5336976911,0.6923159033<br>H,0,-4.4764345867,-2.1259032991,0.6324616884<br>H,0,-5.3521798454,0.1362431572,0.0271075562<br>H,0,-3.274241521,2.8720033476,-0.7717061157
**Anis**
C,0,-1.9697824623,0.0701696922,-0.0140212464<br>C,0,-1.5090433745,-1.2133897919,0.3126228672<br>C,0,-2.4073808158,-2.253387747,0.5643315406<br>C,0,-3.7865801029,-2.0054807836,0.4881134527<br>C,0,-4.2597561787,-0.7344739755,0.1645090013<br>C,0,-3.3494349772,0.3038670997,-0.0866612977<br>O,0,-3.9201477837,1.5352331183,-0.4013965867<br>C,0,-3.0470369325,2.6606309226,-0.6735618554<br>H,0,-1.2572329497,0.8631461363,-0.2057182617<br>H,0,-0.4396787519,-1.3932812952,0.3686142448<br>H,0,-2.0422381396,-3.2433393486,0.8163456279<br>H,0,-4.4942944462,-2.8056181074,0.6816086925<br>H,0,-5.3198634581,-0.518940511,0.0996813232<br>H,0,-3.7144982327,3.4942503213,-0.8885962877<br>H,0,-2.4226986831,2.9018091557,0.1953896814<br>H,0,-2.4049657109,2.4679341142,-1.5417008961
**Guai**
C,0,-2.654333941,-0.4598535828,0.1221298499<br>C,0,-1.8594151526,0.6562631817,-0.1744914112<br>C,0,-0.465455691,0.5576832736,-0.1467871794<br>C,0,0.1510001825,-0.6619037759,0.1788502052<br>C,0,-0.6433143946,-1.7682151193,0.4728684457<br>C,0,-2.0479330196,-1.6702120398,0.4453035552

O,0,-2.8237307903,-2.7801653508,0.7403058047<br>O,0,-0.1841929282,-3.0472320725,0.8141535902<br>C,0,1.2451680366,-3.2810747011,0.8784679686<br>H,0,-3.7366536943,-0.4108693714,0.1079654725<br>H,0,-2.3331256394,1.5991096668,-0.4262399036<br>H,0,0.1491710899,1.4215244591,-0.3763619907<br>H,0,1.2320523365,-0.7341363887,0.1991972716<br>H,0,-2.232177081,-3.5358531132,0.9422829216<br>H,0,1.3565123154,-4.3280254173,1.1578558737<br>H,0,1.7163051502,-3.1036834001,-0.0953089494<br>H,0,1.7139512209,-2.6419742483,1.6357464756
**Ver**
C,0,-0.3462534161,-1.8635419127,0.4615117471<br>C,0,0.3202790086,-0.6535783043,0.206199778<br>C,0,-0.4109957453,0.5000907516,-0.0633351426<br>C,0,-1.8149240051,0.4534521234,-0.0798197333<br>C,0,-2.4848510398,-0.7444666388,0.172661808<br>C,0,-1.7406437694,-1.91853061,0.4470043382<br>O,0,-3.8653922682,-0.8877125165,0.17819769<br>C,0,-4.6802090846,0.2787949099,-0.0991842141<br>O,0,-2.4852641124,-3.0650438539,0.6867796657<br>C,0,-1.7787113302,-4.2981903058,0.9726785008<br>H,0,0.2306703501,-2.7560685499,0.6702818514<br>H,0,1.4047698963,-0.6275147085,0.2211561311<br>H,0,0.0947541517,1.4391901613,-0.2616603277<br>H,0,-2.3757105294,1.3558191301,-0.2902533131<br>H,0,-5.7087997328,-0.0755059345,-0.0424523333<br>H,0,-4.5184995154,1.0669277028,0.6464884906<br>H,0,-4.4807319532,0.6776464983,-1.1014004321<br>H,0,-2.5597350338,-5.0427756071,1.1221471623<br>H,0,-1.1382856115,-4.5967257714,0.1336005244<br>H,0,-1.1718352593,-4.2105345642,1.8822608086
**Eug**
C,0,-4.258017694,4.0694023726,-1.0287615187<br>C,0,-3.048094911,4.2785060666,-1.7055344231<br>C,0,-2.1407470638,3.2307477882,-1.908484211<br>C,0,-2.4659424137,1.9467685238,-1.4247296541<br>C,0,-3.6666649074,1.7410995024,-0.7534277779<br>C,0,-4.5702443395,2.8023655156,-0.5506530645<br>O,0,-5.7637771713,2.5790740153,0.1181068313<br>C,0,-0.8127659924,3.4655575674,-2.6198726079

C,0,-0.53625979,2.4785842255,-3.7307790269<br>C,0,0.5384643344,1.6835235823,-3.7982429078<br>O,0,-4.114975141,0.5247181725,-0.222869887<br>C,0,-3.277439034,-0.6502764596,-0.3686091416<br>H,0,-4.9651658725,4.8746066224,-0.8686775097<br>H,0,-2.8111381886,5.2711218754,-2.0764227473<br>H,0,-1.7747602324,1.1283607707,-1.5910935618<br>H,0,-5.8095376218,1.6344809711,0.3771724715<br>H,0,-0.8186508649,4.4850289196,-3.0313047244<br>H,0,0.0143609363,3.4299034481,-1.8957221258<br>H,0,-1.2913203649,2.4335487561,-4.5161789074<br>H,0,0.6934780629,0.9948145957,-4.6234100004<br>H,0,1.310650274,1.6995874122,-3.0320506329<br>H,0,-3.8244723919,-1.4590871907,0.1146012634<br>H,0,-3.1170385851,-0.888581879,-1.4261938544<br>H,0,-2.3105890272,-0.506333174,0.1270797183
**MEug**
C,0,-1.0977010427,1.1596125287,1.0767812493<br>C,0,-1.5971779226,1.6531548901,-0.1341697668<br>C,0,-1.6030167662,0.8480925527,-1.2792922754<br>C,0,-1.0932159311,-0.4601708297,-1.1891658746<br>C,0,-0.5805915979,-0.9513174671,0.0142704667<br>C,0,-0.5899883432,-0.1356039899,1.1682701858<br>C,0,-2.1238596674,1.3763050682,-2.6114492585<br>C,0,-3.1065336592,0.450629408,-3.2910084998<br>C,0,-2.9544377712,-0.058323112,-4.5195679226<br>O,0,-0.0401001261,-0.5296234059,2.3890530219<br>C,0,-0.5419044595,-1.7563684467,3.0153132719<br>O,0,-0.0354831763,-2.2262990673,0.1610298638<br>C,0,0.0654639404,-3.0782804936,-1.00958588<br>H,0,-1.0860963794,1.770129852,1.9724257304<br>H,0,-1.9856559694,2.6658529366,-0.1832565366<br>H,0,-1.1024742402,-1.0841175474,-2.0750767175<br>H,0,-2.6030324848,2.3494735851,-2.4325793972<br>H,0,-1.2821583136,1.567626386,-3.2930113351<br>H,0,-3.9916665027,0.1961248472,-2.7072521228<br>H,0,-3.6955036175,-0.7147857827,-4.965771464<br>H,0,-2.0837153837,0.1713228615,-5.1299748285<br>H,0,-0.0479039305,-1.7967283053,3.9864887582<br>H,0,-1.6283703628,-1.698561993,3.1543985391<br>H,0,-0.2871558967,-2.6317792292,2.4169456272<br>H,0,0.5488660057,-3.9889330559,-0.6575509891

H,0,-0.9240777058,-3.3171625059,-1.4166035838<br>H,0,0.6786163044,-2.6094556846,-1.7877252621

**iEug**
C,0,-2.328890146,1.9577285987,-0.0282605587<br>C,0,-2.2938941259,0.5615111031,-0.027603379<br>C,0,-1.0715589898,-0.1393388853,-0.0188710326<br>C,0,0.1268454427,0.6122199658,-0.0107308379<br>C,0,0.0882703798,2.0019940319,-0.0114359504<br>C,0,-1.1410323953,2.6850036044,-0.0202051673<br>O,0,-1.1689121449,4.069851846,-0.0208404268<br>O,0,1.1979201899,2.8571026239,-0.0038773494<br>C,0,2.5264464432,2.277308908,0.004609106<br>C,0,-0.9750176999,-1.6079411928,-0.0177593577<br>C,0,-1.9812189095,-2.5017439739,-0.0246431609<br>C,0,-1.7924984507,-3.9927291542,-0.0228650007<br>H,0,-3.267008704,2.5000177956,-0.0349674566<br>H,0,-3.230277045,0.0151534876,-0.0339596583<br>H,0,1.0761332797,0.0879296174,-0.0039173516<br>H,0,-0.2474612358,4.4054471558,-0.0145050442<br>H,0,3.212898102,3.1232294748,0.0086955381<br>H,0,2.6939106619,1.6665659759,-0.8901452427<br>H,0,2.6826869787,1.6670525199,0.9017234494<br>H,0,0.0452807621,-1.9948361626,-0.0104135937<br>H,0,-3.0152372216,-2.1574570346,-0.0320897422<br>H,0,-2.2527863131,-4.4567429633,-0.9067996037<br>H,0,-2.2652927191,-4.4562244383,0.8547179357<br>H,0,-0.7307331393,-4.2632349039,-0.0152461149

**MiEug**
C,0,-2.2511750638,1.2242262549,-0.3202283973<br>C,0,-2.3311786804,-0.1613428199,-0.1834868715<br>C,0,-1.1616682836,-0.9379660684,-0.0781632514<br>C,0,0.0820218919,-0.2723514264,-0.131102875<br>C,0,0.1643411993,1.1134838899,-0.2828823438<br>C,0,-1.0178399623,1.8796571319,-0.3674182338<br>O,0,-1.0324586217,3.2569474748,-0.5808122785<br>C,0,-0.2850361115,4.119621497,0.3393546768<br>C,0,-1.1660431404,-2.4013741711,0.0803311<br>C,0,-2.2339076843,-3.2157677718,0.1664992511<br>C,0,-2.1539934355,-4.7076706519,0.3285970642<br>O,0,1.367785805,1.8139850679,-0.369593881<br>C,0,2.6115843477,1.0668171374,-0.3624509514

H,0,-3.1445433399,1.8328272748,-0.4011950852<br>H,0,-3.3063870874,-0.6338080134,-0.1533964951<br>H,0,0.9877169711,-0.863642463,-0.0607203802<br>H,0,-0.5451723771,5.1357883919,0.0419212165<br>H,0,0.7881338073,3.9519475181,0.2458870738<br>H,0,-0.6039439386,3.9425748135,1.3736270497<br>H,0,-0.1757593029,-2.856168175,0.132854624<br>H,0,-3.2401629461,-2.7999161677,0.1197068499<br>H,0,-2.6568176611,-5.0383271441,1.2483943672<br>H,0,-1.1148713787,-5.0524725548,0.3698416589<br>H,0,-2.6499954031,-5.2283030469,-0.5027665312<br>H,0,3.3940552666,1.8162466459,-0.4766209273<br>H,0,2.6522875511,0.358342378,-1.1978044553<br>H,0,2.7496325785,0.5290379984,0.5831030258
**Est**
C,0,-1.1744036132,1.2072712749,1.04561661<br>C,0,-1.5300476458,1.6937245153,-0.2110635108<br>C,0,-1.6185410961,0.8412554336,-1.3269193719<br>C,0,-1.3437160155,-0.5206677926,-1.1373978518<br>C,0,-0.9845068281,-1.0270626831,0.1178905547<br>C,0,-0.8995827578,-0.1572643005,1.2127452833<br>C,0,-1.9856607336,1.383692872,-2.7036480734<br>C,0,-3.1004955722,0.6182642583,-3.3790717963<br>C,0,-3.0081455678,0.0283295552,-4.5768489631<br>O,0,-0.5548995234,-0.5485844353,2.5051105866<br>C,0,-0.259341599,-1.9463072796,2.7536449431<br>H,0,-1.1059490496,1.8596779712,1.908410716<br>H,0,-1.7422496755,2.7527250011,-0.3299346556<br>H,0,-1.4170829042,-1.2003976524,-1.9812133693<br>H,0,-0.7789828916,-2.0852527673,0.2259036004<br>H,0,-2.2826348968,2.4368942583,-2.5934394187<br>H,0,-1.1019740794,1.3806456992,-3.3583181976<br>H,0,-4.0336721117,0.5574554997,-2.8182745675<br>H,0,-3.8429724274,-0.5062887093,-5.0200292712<br>H,0,-2.0922156261,0.0632062505,-5.1627851491<br>H,0,-0.0238122416,-2.0044491065,3.8158329566<br>H,0,-1.1241638347,-2.5834281018,2.5320854491<br>H,0,0.6029116911,-2.2833097608,2.1655194963
**Aneth**
C,0,-1.9822312773,1.7356679606,-0.4204799486<br>C,0,-1.5517135123,0.4456019886,-0.0905924113

C,0,-2.4565338101,-0.6031962714,0.1545761038<br>C,0,-3.8363243799,-0.3063492035,0.0552639562<br>C,0,-4.2795013869,0.9688664697,-0.2711594302<br>C,0,-3.3535615129,1.9980339899,-0.5111108357<br>C,0,-1.9346848661,-1.9353224977,0.4965852382<br>C,0,-2.6361945295,-3.0529081896,0.7620434851<br>C,0,-2.0213056677,-4.380927981,1.1049040946<br>O,0,-3.9022739302,3.236654448,-0.8302573456<br>C,0,-3.0081360404,4.3484440597,-1.0922040289<br>H,0,-1.2487481787,2.5120222854,-0.6007731322<br>H,0,-0.4851980508,0.249070982,-0.0219946628<br>H,0,-4.5707034662,-1.0840950821,0.2360229234<br>H,0,-5.3361714939,1.197779785,-0.3476157549<br>H,0,-0.8461145775,-1.9986734176,0.5328207249<br>H,0,-3.725625913,-3.0315335245,0.7361130576<br>H,0,-0.9270321861,-4.3268187101,1.111343269<br>H,0,-2.3479970164,-4.7310366606,2.094396768<br>H,0,-2.316880918,-5.1578153136,0.3855608659<br>H,0,-3.6593763888,5.1928152359,-1.3153996156<br>H,0,-2.3911589292,4.5792598358,-0.2154112031<br>H,0,-2.3594829681,4.1444598113,-1.9526331178
**1,8-cineole**
C,0,-1.0032386424,0.2131703135,-0.6083080726<br>C,0,-1.2099693674,-1.2952632815,-0.3564453419<br>C,0,-0.7576707411,-1.6565635269,1.0906682517<br>C,0,-0.1660576026,-0.3936536593,1.766277237<br>C,0,1.0012651752,0.1775172435,0.9098833725<br>O,0,0.4500709304,0.5072597256,-0.4477393794<br>C,0,2.1377525971,-0.8347351114,0.6934198411<br>C,0,1.5827075231,1.4808677885,1.4811179803<br>C,0,-1.3360636607,0.6203861853,-2.0402855243<br>C,0,-1.7715947521,1.0455051524,0.4397342851<br>C,0,-1.3099198601,0.6462449613,1.8738538549<br>H,0,-0.6253874993,-1.8518266688,-1.1024597986<br>H,0,-2.2683147385,-1.5460845303,-0.5180592899<br>H,0,-0.0303522004,-2.4750744333,1.0783291771<br>H,0,-1.6092461841,-2.0044573454,1.6891574602<br>H,0,0.2223969453,-0.641344476,2.768470726<br>H,0,2.9167994089,-0.3851006078,0.0652862754<br>H,0,1.7841011395,-1.7427468077,0.1956253564<br>H,0,2.5901834593,-1.1154983112,1.6537120952<br>H,0,2.3818657527,1.8465685466,0.8244249741<br>H,0,2.0095955635,1.3067437727,2.477678225

H,0,0.8234680578,2.2649921652,1.5589311231
H,0,-1.1297797631,1.6866694867,-2.1947979235
H,0,-2.3973542742,0.4372848219,-2.2503822547
H,0,-0.7355051214,0.0429032158,-2.753888856
H,0,-2.851140811,0.8829013498,0.3083281852
H,0,-1.5759936925,2.1093441125,0.2447483202
H,0,-0.9901793584,1.5276980039,2.4394580019
H,0,-2.1369352835,0.1963739148,2.4378216984

Figure S1. GC-MS chromatograms of bay laurel EO samples. Peaks 1-7 were cross-referenced against the NIST mass spectral library (version 2.0f, 2008) and assigned to: (1) limonene; (2) 1,8-cineole; (3) linalool; (4) terpinen-4-ol; (5) α-terpinyl acetate; (6) MEug; (7) Eug.

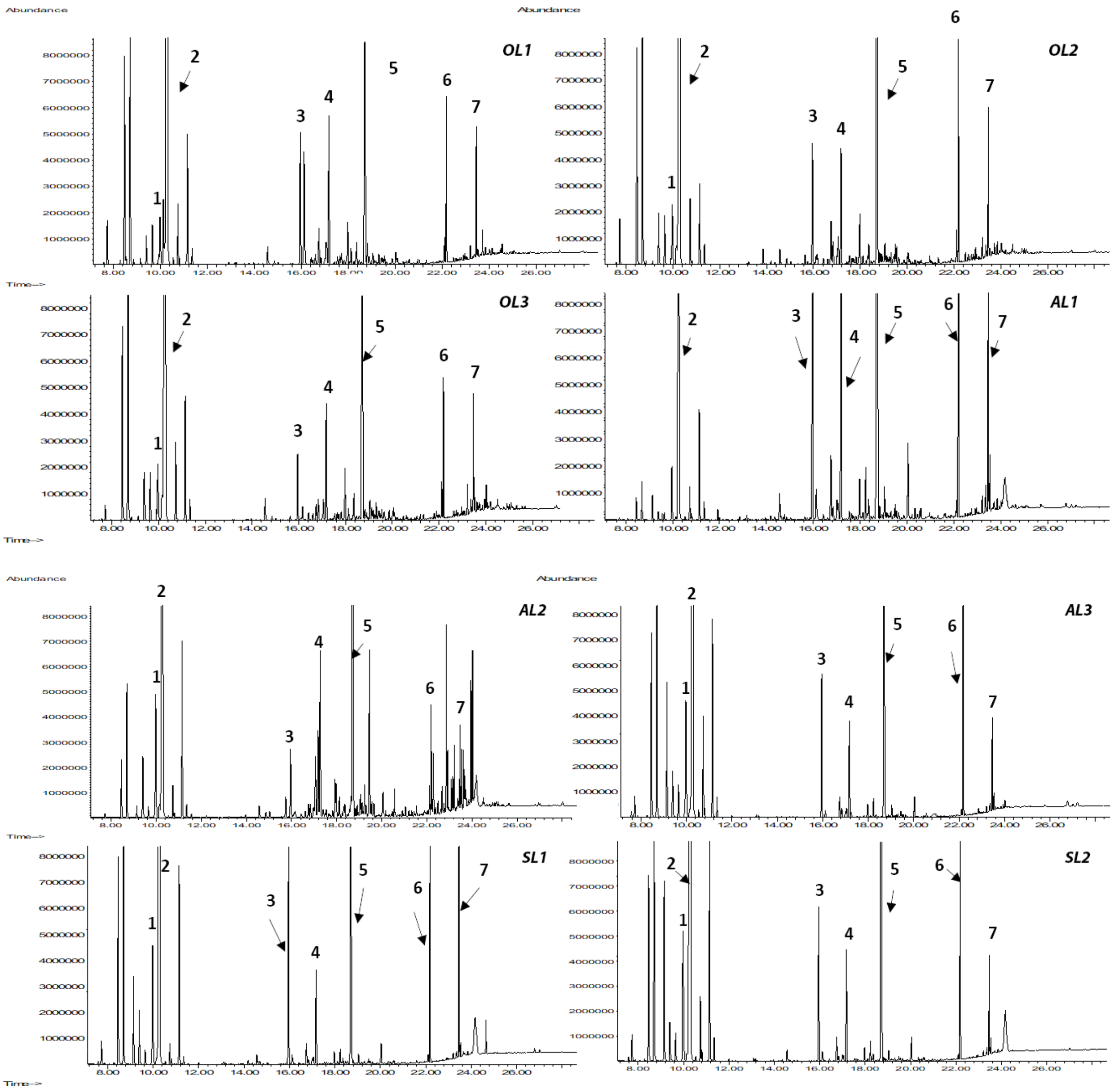

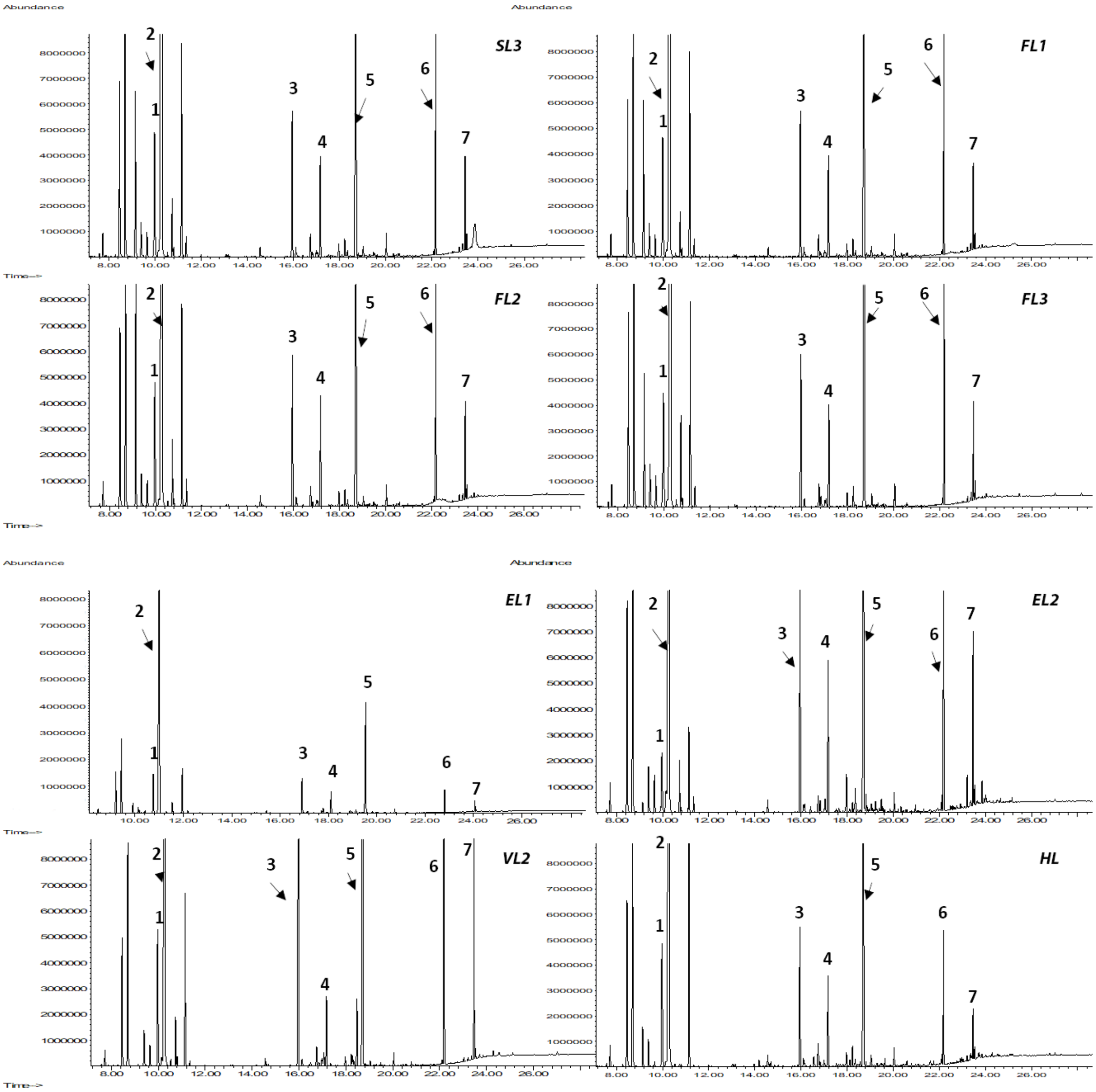

Abundance
SL3
FL1
FL2
FL3
EL1
EL2
VL2
HL
1
2
3
4
5
6
7
Time-->

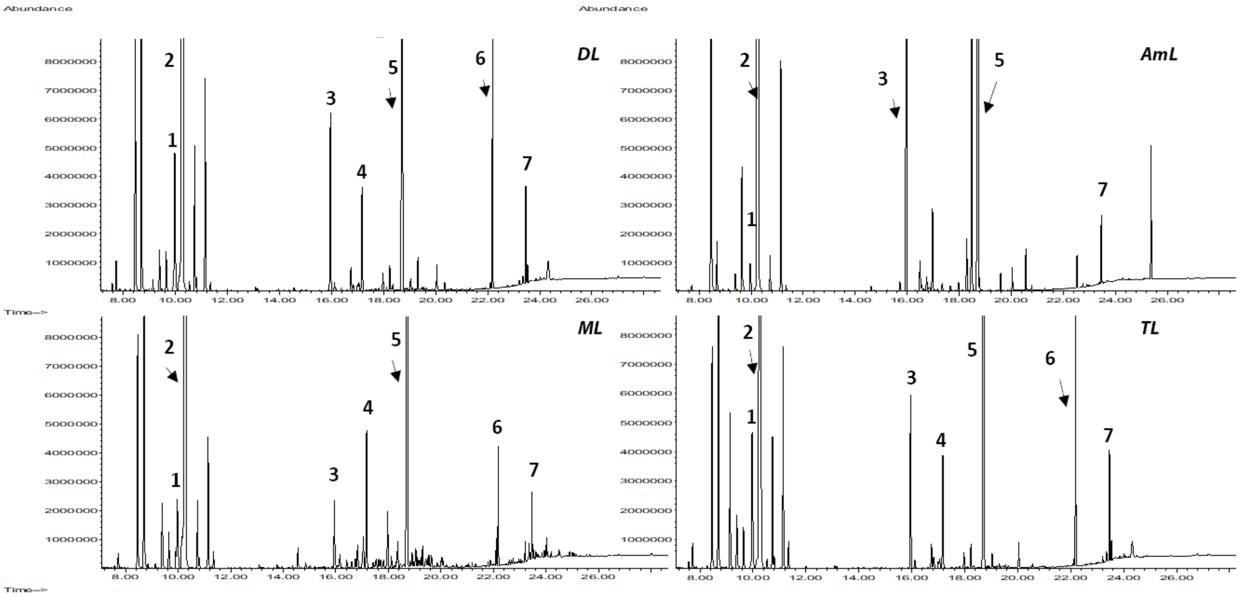

Abundance
Time-->
DL
AmL
ML
TL
